# Teach and Playback Training Device for Minimally Invasive Surgery

**DOI:** 10.1155/2018/4815761

**Published:** 2018-01-10

**Authors:** Sriram Garudeswaran, Sohyung Cho, Ikechukwu Ohu, Ali K. Panahi

**Affiliations:** ^1^Industrial Engineering, Southern Illinois University Edwardsville, Edwardsville, IL 62026, USA; ^2^Industrial Engineering, Gannon University, Erie, PA 16541, USA

## Abstract

Recent technological progress offers the opportunity to significantly transform conventional open surgical procedures in ways that allow minimally invasive surgery (MIS) to be accomplished by specific operative instruments' entry into the body through key-sized holes rather than large incisions. Although MIS offers an opportunity for less trauma and quicker recovery, thereby reducing length of hospital stay and attendant costs, the complex nature of this procedure makes it difficult to master, not least because of the limited work area and constricted degree of freedom. Accordingly, this research seeks to design a Teach and Playback device that can aid surgical training by key-framing and then reproducing surgical motions. The result is an inexpensive and portable Teach and Playback laparoscopic training device that can record a trainer's surgical motions and then play them back for trainees. Indeed, such a device could provide a training platform for surgical residents generally and would also be susceptible of many other applications for other robot-assisted tasks that might require complex motion training and control.

## 1. Introduction

This past decade has seen an undeniable turn toward new approaches to minimally invasive surgery (MIS), fueled by modern enhancements to the designs of surgical instruments as well as to techniques for vision and image processing. MIS procedures are generally carried out through a half-inch incision (or multiple such incisions), offering patients benefits in comparison to traditional open surgery that include alleviated trauma level, brief hospital stay, and higher-quality care [1, 2]. Yet MIS is more challenging for the surgeon than open surgery is [3, 4]. Indeed in MIS, visual feedback from the operating area is available only by means of an endoscopic camera. Accordingly, rather than using direct observation, surgeons use a two-dimensional (2D) screen to monitor operations—an approach that deprives them of means for gauging depth. What is more, because of kinematic constraints at incision points, instruments' motions are restricted. Diminished visual and tactile perception and lack of scope for dexterity notwithstanding surgical approaches are trending toward minimally invasive techniques, seeking to offer patients additional benefits even if doing so increases stress on surgeons [5]. Accordingly, means of resolving these technical issues are in high demand, especially for complex tasks such as suturing. In particular, training in MIS procedures is required to ensure safe practice in a finite working space offering reduced tactile feedback. Thus recent studies have indicated a need for task-oriented tests that encompass the complexities of MIS as they relate to visual, spatial, cognitive, perceptual, and motor factors—which are also critical for investigating, classifying, and designing training programs for surgeons at all levels with a view to instituting preferred models for structured, quantitative, and objective education and assessment [6–9]. This research, then, seeks to meet these needs by developing a physical Teach and Playback training device.

Use of MIS improves surgical results while diminishing recovery time, but it has also complicated surgical tasks, increasing surgeons' stress levels as the intricacy of their work also increases. Surgical ergonomics research has naturally followed that has sought ways of reducing stress, improving skills, and assessing quantitative skills. This last object seeks to uncover vital information relating to a trainee surgeon's level of expertise as well as the effectiveness of the training process itself. Moreover, it can provide timely performance feedback to trainees at all levels, greatly assisting in competence reviews. Such an approach can drastically reduce training time—and researchers and expert surgeons, for their part, can use assessment information to aid their own research and analysis while improving the ergonomics of surgery.

Historically, assessment of surgical skills has been subjective, depending on direct observation, global assessments, and the use of checklists [10]. As part of this assessment, experienced surgeons observe trainees during MIS—a time-consuming approach that is also resource- and effort-intensive. Objective assessment, however, poses its own challenges, arising from differences in patients, surgical teams, and operational setup, among other factors [11]. In recognition of this, efforts at improving and formulating objective evaluation approaches have proliferated during the past few years [12]. As part of this effort, MI researchers, along with researchers in other areas, have collaborated to develop ways of objectively assessing surgical skills. Yet an extensive review of the literature reveals that a standalone solution to these challenges is not yet available—although use of virtual reality (VR) systems has shown some promise [11].

Patients increasingly prefer MIS, but the number of well-trained surgeons is falling short of the demand—partly owing to a lack of MIS equipment and partly because of the prohibitive cost of such equipment. As a result, little equipment is available, whether for the operating room (OR) or for training. What is more, no quantitative standard exists for real-time assessment of surgical skills. Accordingly, this research aims to provide a low-cost and portable Teach and Playback laparoscopic training device able to record a trainer's surgical motions and play them back for trainees. Using such a method would greatly speed learning time by providing ready access to expert practice, thereby avoiding subjective (“self-discovery”) approaches to learning complex skills.

The Teach and Playback training device prototype offers a robot arm that enjoys 6 degrees of freedom (6DoF). Although, traditionally, DC servomotors comprise three terminals (positive, negative, and signal), a generally unused terminal within the servomotor's housing can be harnessed as a potentiometer, with the shaft of the servomotor itself acting as a variable rheostat. Accordingly, when key-framing motions use the Teach and Playback device, the 6DoF robot arm is able to encode angular information derived from the shafts of its six different servomotors, which also act as potentiometers, for processing and analysis. This simple design choice serves as the kernel of the idea of the Teach and Playback device and its construction: the device records a robot's end-effector motion path, physically key-framing it so as to reproduce precisely the same motions. Thus sensors eliminate complex inverse kinematic equations from the calculations.

## 2. System Structure

**Figure 1:**
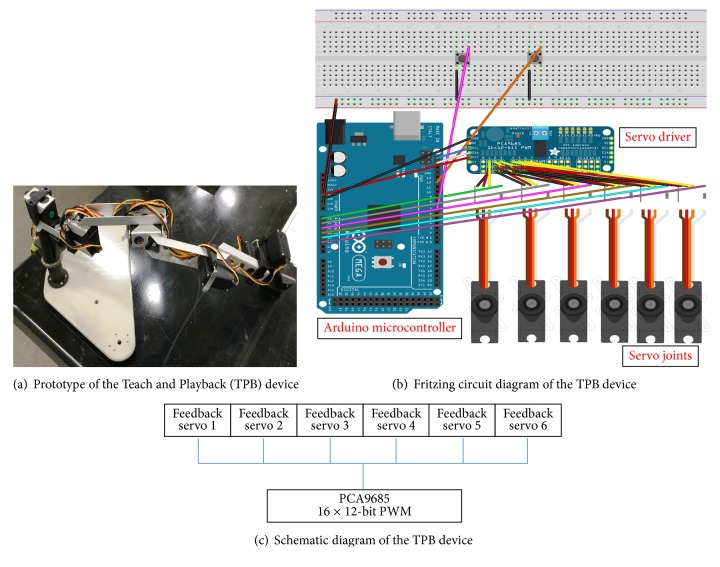
The Teach and Playback device. (a) 6DoF Robot Arm. (b) Fritzing circuit diagram. (c) Fritzing block diagram.

The system has a 6 DoF (degree of freedom) robotic construction that itself is built up out of six servomotors, mechanically connected to each other by aluminum links that form the robot's armature.  Figure 1 shows the system structure. In its offline (nonexcited) mode, each servo also acts as a potentiometer so that, during physical key-framing, the armature with its six servomotors, all now acting as potentiometers, emits instantaneous angular information from the shaft of the servomotors for processing and analysis. Thus the sensors eliminate the need to solve complex inverse kinematic equations.

The signal and potentiometer terminals of each servo connect, respectively, to the “digital” output pins of a servo driver and the “analog” input pins of an Arduino microcontroller. These integrated circuits (ICs) exert control, route instructions, and allow receipt of data. The Arduino microcontroller is itself connected by universal serial bus (USB) to a PC or workstation, communicating serially in duplex (2-way) mode. Thus physically key-framed information is transmitted to a database hosted on the workstation for storage and processing—and can also be transmitted on demand to the Arduino microcontroller over the serial duplex channel. This is the whole of the system. The Adafruit® 16-channel 12-bit PWM/servo driver, built using the PCA9685, drives up to 16 servos over I^2^C, making use of only two pins. The onboard PWM controller drives all 16 channels simultaneously, requiring no additional Arduino processing overhead. For purposes of expansion, up to 62 drivers can be chained to control up to 992 servos, all using the same two pins: the SDA (data) and SCL (clock) ports. Servo motors allowing for precise control of angular position, velocity, and acceleration are used, coupled to sensors for position feedback.

The Parallax Data Acquisition tool, commonly known as PLX-DAQ, is a software add-in for Microsoft Excel that acquires up to 26 channels of microcontroller data through serial communication, arranging the data into columns. The PLX-DAQ thus offers an easy means of conducting spreadsheet analysis of data collected in the field, along with laboratory analysis of sensors and real-time equipment monitoring.

### 2.1. Servo Modification

Traditionally, a servomotor accepting direct current (DC) input comprises three electrical terminals: positive, negative, and signal. In general, however, there is one more terminal for the IC to protect the servomotor from potential overcurrent faults and shocks. Tapping into this terminal allows the addition of a fourth and new connection, this one serving as a potentiometer, with the servo motor itself acting as a variable rheostat in its offline mode.

Accordingly, during physical key-framing of motions using the Teach and Playback device in its offline mode (i.e., when the servos are not driven), the armature and its six different servomotors, each now acting as a potentiometer, emits moment-by-moment angular information from the shaft of the servomotor for processing and analysis. This simple concept, unique to this project, serves as the essential concept around which the Teach and Playback device is built.

### 2.2. Robot Kinematics

The kinematic equations for a robot's series chain are obtained using a rigid transformation *Z* to characterize the relative movement allowed at each joint and using a separate rigid transformation *X* to define the dimensions of each link. The resulting sequence is one of rigid transformations, alternating joint and link transformations from the base of the chain to its end link, the position of which is calculated as follows:

(1) $$\begin{array}{c} \left[\;T\;\right]=\left[\;Z_{1}\;\right]\left[\;X_{1}\;\right]\left[\;Z_{2}\;\right]\left[\;X_{2}\;\right]\cdots \left[\;X_{n-1}\;\right]\left[\;Z_{n}\;\right], \end{array}$$

where *T* is the transformation locating the end link.

These equations represent the kinematics equations of the serial chain [13].

In 1955, Denavit and Hartenberg introduced a convention for defining joint matrices *Z* and link matrices *X* so as to standardize the coordinate frame for spatial linkages [14, 15]. This convention positions the joint frame such that it consists of a screw displacement along the *Z*-axis:

(2) $$\begin{array}{c} \left[\;Z_{i}\;\right]={Trans}_{Zi}\left(\;d_{i}\;\right){Rot}_{Zi}\left(\;\theta_{i}\;\right) \end{array}$$

and positions the link frame such that it consists of a screw displacement along the *X*-axis:

(3) $$\begin{array}{c} \left[\;X_{i}\;\right]={Trans}_{Xi}\left(\;a_{i,i+1}\;\right){Rot}_{Xi}\left(\;\alpha_{i,i+1}\;\right). \end{array}$$

Using this notation, each transformation link going along a serial chain robot can be described by the coordinate transformation

(4) Ti i−1=ZiXi=TransZidiRotZiθi·TransXiai,i+1RotXiαi,i+1,

where *θ*_*i*_, *d*_*i*_, *α*_*i*,*i*+1_, and *a*_*i*,*i*+1_ are Denavit-Hartenberg parameters.

### 2.3. Kinematic Analysis

Through kinematic analysis, the designer can discover the position of each component of the mechanical system—data needed for subsequent dynamic analysis and control paths. Inverse kinematics exemplifies the analysis of a constrained system of rigid bodies, or a kinematic chain, such that a robot's kinematic equations can be used to define loop equations for a complex articulated system, which are nonlinear constraints on the configuration parameters of the system. The independent parameters in these equations are known as the system's degrees of freedom. Although analytical solutions to the inverse kinematics problem exist for a wide range of kinematic chains, computer modeling and animation tools often use Newton's method of solving nonlinear kinematics equations. Other applications of inverse kinematic algorithms include interactive manipulation, animation control, and collision avoidance.

## 3. System Programming

### 3.1. Communication Interface and Hardware Programming

Extensive programming was used to establish the communication interface and to program the microcontrollers to communicate among all interfaces in the control system, allowing for the sending, receiving, and routing of angular values as well as the use of a user interface. Communications to and from the Arduino microcontroller are conducted using the Arduino programming language, which resembles the C programming language and features C-style constructs. It is used to read and route, via the microcontroller, potentiometer values to a local computer database. Communication between potentiometers and Arduino microcontroller is established by means of the I^2^C communication protocol, whereas communication between Arduino microcontroller and computer is by USB.

### 3.2. User Interface and Software Programming

**Figure 2:**
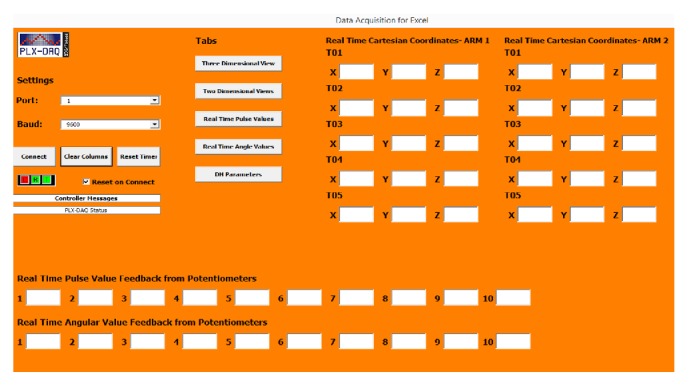
Graphical User Interface (GUI) for the “Teach and Playback” device system.

**Figure 3:**
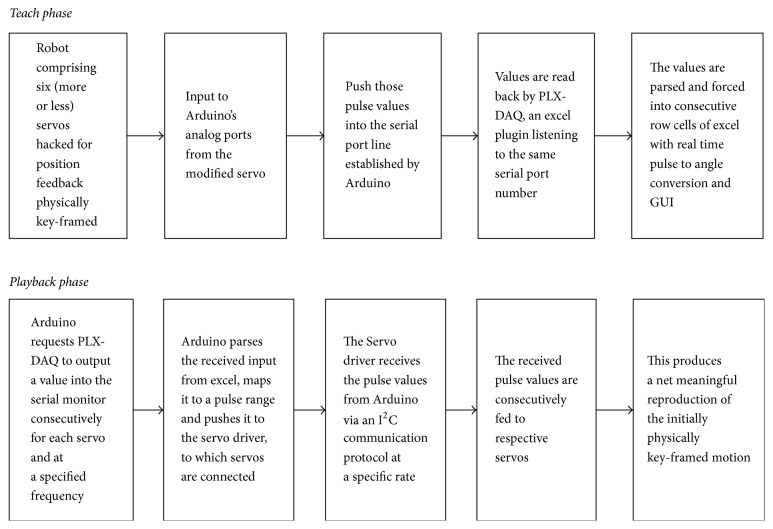
Logical activity flow during different phases of system operation.

The PLX-DAQ Microsoft Excel plugin is coded in the backend using Visual Basic for Applications (VBA), as is the custom-designed user interface, which can be tweaked to populate angular values and graphical illustrations in real time as shown in Figure 2.  Figure 3 illustrates the sequence of* Teach *and* Playback *actions.

## 4. Data Validation

**Figure 4:**
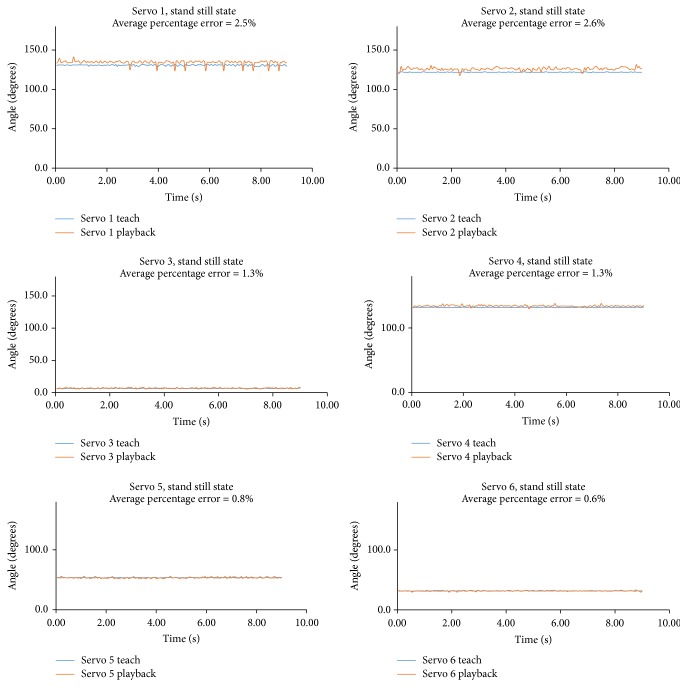
Fixed-position servo values during the “teach” and “playback” stages.

**Figure 5:**
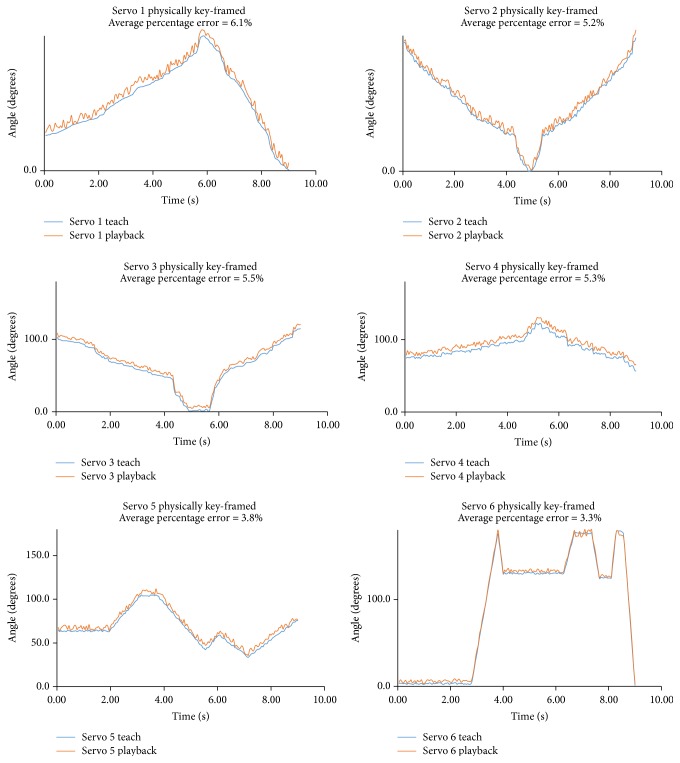
Servo values during the “teach” and “playback” stages of a surgical exercise (peg transfer).

Rather than physically key-framing motions into the Teach and Playback device, the device is left energized, with values recorded by the servos during the “teach” and “playback” phases recorded and compared with one another.  Table 1 and Figures 4 and 5 present observations from each servo, 1 through 6, during the Teach and Playback device as already discussed, when no motion is physically key-framed and when it is in its standstill state, with pulse values recorded at 40 Hz for 9 seconds. Angular values and percentage error were also computed for the pulse values, during the “teach” and “playback” phases.  Table 1 lists average pulse values, percentage error, and standard deviation for servos 1–6 during the “teach” and “playback” phases.

The foregoing values show that the average error percentage calculated at the sixth joint or the sixth servo is far less than the average error percentage calculated at the first servo. Another interesting observation may also be made: the average error percentage increases from the sixth servo to the first. A similar pattern is also observed in the standard deviation calculated for each of the servos. As with the computed error percentages, the standard deviation possesses a higher value at the sixth servo than at the first servo, and the standard deviation value continually increases from the sixth servo joint to the first.  Figure 4 shows the servo values at a fixed position during the “teach” and “playback” stages.

This behavior comports with existing knowledge: The load on the sixth servo is far less than that endured by the first, for the load on the sixth servo is directly applied to the fifth servo, taking weight off the sixth servo itself, and so on to the first. Thus the first servo, being in base position, bears the highest load of all. Accordingly, error and deviation of output from input are greatest at the first servo but decrease through to the sixth servo. In this manner, theoretical understanding is reinforced by calculated observations.

Figure 5 depicts the servo values in the “teach” and “playback” stages during a surgical exercise (peg transfer), during which the average error percentage calculated at the sixth joint or the sixth servo is noticeably less than that at the first servo. This echoes previous observations about the fixed position, which can be attributed to the difference in load endured by servos in various positions.

## 5. Conclusions

### 5.1. Design Contributions

In this study, a novel Teach and Playback training device was developed—essentially a 6 DoF robot that can be used to train novice surgeons to perform minimally invasive surgery by first recording the surgical motions of a trainer and then playing back those same motions for trainees. This device thus seeks to address the need for a quantitative standard for real-time surgical skills assessment.

In developing an interactive communication protocol for high-speed duplex communication, this study sought to seamlessly connect such a training device to a computer workstation or laptop by means of an open-source microcontroller, making use of the ubiquitous Microsoft Excel as a database for all physically key-framed angular information. Such a method combines visually pleasing access to raw data with the facility for instantaneous pattern recognition and identification, along with unlimited storage capacity and organized data storage.

This research also explains how a simple servo hack may accommodate a fourth terminal, enabling the servomotor to act as a potentiometer in its standalone state and thereby form the entire basis for development of the low-cost Teach and Playback device in question. Notably, this research uses readily available and low-cost components and devices, together with open-source architecture and freeware, to facilitate construction.

During error analysis performed on the pulse and angular values recorded from the joints of the Teach and Playback device for each of the six servos in its standstill state, as well as when a practical action (peg transfer) was performed using the device, with motions physically key-framed into the device, observed percentage error values were within reasonable and permissible levels. Indeed, the average percentage error and standard deviation values recorded aligned with the system's theoretically expected behavior.

### 5.2. Recommendations for Future Research

The proposed design paradigm should thus be applied to an industrial-grade servo, allowing its performance to be gauged in a real-world situation. Also, the use of a more robust GUI-based middleware software package would enhance operation during the “playback” phase, making it smoother and more accurate. Blender [16] can be considered, a professional open-source 3D computer graphics software product was used to create animated films, visual effects, art, 3D-printed models, interactive 3D applications, and video game assets. Importantly, the game engine within this software application could be used to build a virtual model of the Teach and Playback device's entire armature. This same engine also flaunts an inverse kinematics system that could be used to compute joint positions and servo angles at a moment's notice and with far greater precision and accuracy. Accordingly, using this software would diminish the effects of rounding and would reduce the effects of other sources of potential noise arising during communication. Continuation of research in this vein could thus make use of the many features available in more sophisticated software packages to enhance precision, boost accuracy, and improve visualization.

## Figures and Tables

**Table 1:** Mean pulse and standard deviation values in the “teach” and “playback” phases, and average percentage errors in the stationary and physically key-framed states.

	Teach phase (pulse value)		Playback phase (pulse value)		Average error (%)	
	Mean	St. dev.	Mean	St. dev.	Stationary	Key-framed
Servo 1	296.5	1.22	302.6	4.88	2.5	6.1
Servo 2	281.2	0.42	288.4	3.25	2.6	5.2
Servo 3	81.3	0.49	81.9	1.18	1.3	5.5
Servo 4	298.4	0.58	302.3	1.76	1.3	5.3
Servo 5	162.6	0.48	162.5	1.68	0.8	3.8
Servo 6	124.8	0.66	124.8	0.99	0.6	3.3
